# The effect of slow breathing in regulating anxiety

**DOI:** 10.1038/s41598-025-92017-5

**Published:** 2025-03-11

**Authors:** Qian Luo, Xianrui Li, Jia Zhao, Qiu Jiang, Dongtao Wei

**Affiliations:** 1 Key Laboratory of Cognition and Personality (SWU), Ministry of Education, Chongqing, 400715 China; 2 Faculty of Psychology, Southwest University (SWU), Beibei District, Chongqing, 400715 China; 3 Shandong Daizhuang Hospital, Jining, Shandong 272051, China

**Keywords:** Respiration rate, Slow breathing, Uncertainty, Anxiety, EEG, Respiration, Emotion

## Abstract

Anxiety is an interactive disorder of the mind and body, characterized by excessive worry about uncertain future events and a dysfunction of the autonomic nervous system. Previous studies have shown that slow, deep breathing can reduce physical tension, and anxiety. Although we know that slow and deep breathing techniques can effectively regulate anxiety and other emotions, the psychological and neurophysiological mechanisms of slow breathing on anxiety have not been systematically explored. In the study, we combined the paced breathing task with the threat uncertainty task for the first time to investigate the role of slow breathing in regulating anxiety. Here we investigated this question, using Spectral analysis and Time-frequency domain of EEG to assess brain activity relating respiratory rate and the mechanism of respiratory rate impact on the anxious. Twenty-seven individuals participated in the experiment, which followed a 2 (respiratory rate: fast breathing, slow breathing) × 2 (certainty: certain, uncertain) within-subjects design. The results of showed that: (1) Slow breathing effectively reduced anxiety, the valence and arousal are lower under the slow breathing. (2) The EEG of fast and slow breathing showed different characteristics. There is an overall increase in power during slow breathing in the delta, theta, alpha and beta bands.(3) The interaction of respiratory rate and certainty were closely related to beta. In the uncertain, beta power decreased with slow breathing but increased with fast breathing.

## Introduction

Anxiety disorders are the most common mental illness in children and adolescents, with prevalence rates as high as 20%^1^. Anxiety disorders are typically characterized by persistent and excessive worry, often accompanied by physical and cognitive symptoms that can lead to dysfunction^2^. Anxiety in moderation is useful. However, when anxiety is severe, it can become chaotic and therefore counterproductive. Having information about a potential future event reduces the anxiety associated with it.However, there is often uncertainty as to whether the upcoming aversion event will actually happen, and how dangerous or negative it will be. Uncertainty about a possible future threat disrupts our ability to avoid it or to mitigate its negative impact and thus results in anxiety^3^, that has historical roots in animal studies of stress response and fear learning, as well as in previous influential models of anxiety pathology. Scholars provided evidence that anticipation about potential future threats induce anxiety in multiple levels, which range from affective and cognitive to behavioral manifestations^3–5^.Anticipatory anxiety is defined as the time between warning and stimulus^6^. In real life, it is frequently characterized by potential unfavorable clues that signify future threats, such that people with anticipatory anxiety remain in a state of stress, including physiological arousal, cortical activation, and cognitive bias^5,7,8^. Constant stress can take a toll on one’s body and mind. Uncertainty is a constant in everyday life, making it increasingly vital to address the negative expectations it brings.

New evidence has demonstrated that respiratory rhythms exert surprising, substantive influences on perception, emotion, and cognition^9^. Breathing is an integral component of interoceptive processing, that is, the state of the physiological condition of the body^10^. Respiration is unique compared with other systems (e.g. gastrointestinal) insofar as conscious regulation can immediately impact respiratory processes. Instructed breathing patterns are widely applied to treat a variety of complaints and conditions, e.g., pain, stress, post-traumatic stress disorder^11–13^. Most often, these patterns involve a voluntary reduction of breathing frequency, which is assumed to increase parasympathetic activity. Slow breathing at 4.5 to 6.5 breaths per minute (coherent or resonance breathing) has been shown to optimally balance sympatho-vagal stress response for most adults^14–17^.Heart rate variability(HRV), the beat-to-beat variation in either heart rate or the duration of the R-R interval–the period, has become a popular clinical and investigational tool. HRV is an index of cardiac autonomic activation as well as an outcome variable in breathing training or HRV biofeedback research^18,19^.

The relationship between breathing and electroencephalogram (EEG) activity was first mentioned by Hobson. The study found that with the change of frog’s state from rest to activity, EEG also changed from low frequency fast wave mode to high frequency slow wave mode, and the degree of synchronization of EEG was proportional to the respiratory frequency^20^. A sthdy described the oscillatory activity of the human EEG during the breathing cycle, and found that the amplitude of the EEG curve increased during inhalation, while the EEG curve showed the opposite trend during exhalation. A cyclic pattern of electrical activity in the brain can be observed during sleep as breathing changes^21^. Later, it was found that different modes of nasal breathing (including left nostril inhalation and right nostril exhalation, and vice versa) increased power in the beta and alpha bands^22^.The results of electroencephalogram (EEG) studies on breathing focused meditation activities indicate an increase in alpha and theta power in the posterior brain region and the frontal region^23,24^. An increase in frontal theta may indicate a need for cognitive control and call on other brain regions^25^. Recently, one study compared Heartbeat Evoked Potential (HEP)activity for heartbeats occurred during inhalation and exhalation at rest. the found higher HEP amplitude during exhalation, compared to inhalation, over fronto-centro-parietal areas. This suggests increased brain-heart interactions and improved cortical processing of the heartbeats during exhalation^26^.There is growing evidence that breathing directly effects on neural oscillations in various brain regions^27^. Previous studies have primarily focused on the characteristics of brain activity during exhalation and inspiration. However, given the benefits of slow breathing for emotional regulation, it is essential to also consider the characteristics of brain activity during slow breathing.

Anxiety are the psychological symptoms typically associated with autonomic overactivity, subjectively, the individual feels nervous and flushed, heart palpitations, shortness of breath, increased sweating^28^. Changes in breathing can be both the consequence of an increased level of anxiety as well as the source of threat experienced by the individual, which, in turn, leads to increased anxiety. Thus, assessing breathing might be a useful physiological marker of the level of anxiety but can also serve as an experimental tool to influence anxiety levels^29^. A review by Weng examined studies on the manipulation of breathing^30^. It suggests that slow breathing may activate cardiopulmonary pressure receptors, resulting in reduced reflexive sympathetic nerve activity and subsequently lower anxiety levels. However, the exact process remains unclear.

This study combined paced breathing with the threat uncertainty task^31^ to create a new experimental paradigm. The aim was to induce anticipatory anxiety and investigate whether slow breathing is beneficial in reducing responses (valence, arousal) associated with anticipatory anxiety. After participants performed breathing exercises, we investigated the genesis and development of biased estimates of emotional pictures under conditions of uncertainty. We continuously monitored participants’ self-reported scores of emotional pictures^32^and EEG measurements of neuronal activity during the experiment. On the basis of conceptualizing uncertainty as an anxiety state, we expected to observe biases related to uncertainty in post-experiment estimates, where uncertain cues would be overly associated with negative pictures. Across species, physiological responses to threats are heightened when there is uncertainty about their nature, probability, or timing^33–37^.Furthermore, aversive events that are not fully predictable have a greater negative impact on mood, state anxiety and physiological indices of reactivity than those that are fully predictable^34,36,37^. We hypothesized that: (1) Slow breathing has a significant regulating effect on anticipatory anxiety. Compared with fast breathing, individuals under slow breathing conditions exhibit lower valence and arousal in response to negative images; (2) Uncertainty can increase anticipatory anxiety, and individuals show more valence and arousal to pictures presented after uncertain cues; (3) The electrical activity of the brain varies significantly at different breathing rates, exhibiting distinct characteristics between fast and slow breathing patterns.

## Materials and methods

### Participants

Twenty-seven college students were recruited for this study, but two participants lacked complete EEG data. The final sample included 25 healthy female participants from Southwest University who took part in the study. The participants were 18–26 years old, with a mean age of 20.72 years (SD = 2.324). All individuals signed a written informed consent form. According to self-reports, all participants were right-handed, had no psychiatric or neurological diseases, and had normal or corrected-to-normal eyesight. This study was approved by the Ethics Committee of the Department of Psychology, Southwest University and all experiments were performed in accordance with relevant guidelines and regulations.

## The paced breathing-threat uncertainty task and procedure

The experiment took place in a tranquil room with a suitable temperature. Upon their arrival at the laboratory, participants were given some time to rest. Following this, they received instructions regarding the tasks they were expected to perform and engaged in practice for the experimental procedures. Once they were comfortable with the tasks, the experiment was carried out in four blocks, each consisting of 15 trials. Participants had the opportunity to take a brief rest of 1–2 min after completing each block. Ultimately, all participants finished the trials by the conclusion of the study.

Twenty-seven individuals participated in the experiment, which followed a 2 (respiratory rate: fast breathing, slow breathing) × 2 (certainty: certain, uncertain) within-subjects design. In the paced breathing task, where they followed audio-guided breathing exercises. This audio was produced by an instructor with nine years of yoga teaching experience. The breathing conditions were categorized into fast breathing (both the exhalation time and the inhalation time were 2 s) and slow breathing(both the exhalation time and the inhalation time were 6 s), each breathing exercise lasted for 30 s.

**Fig. 1:**
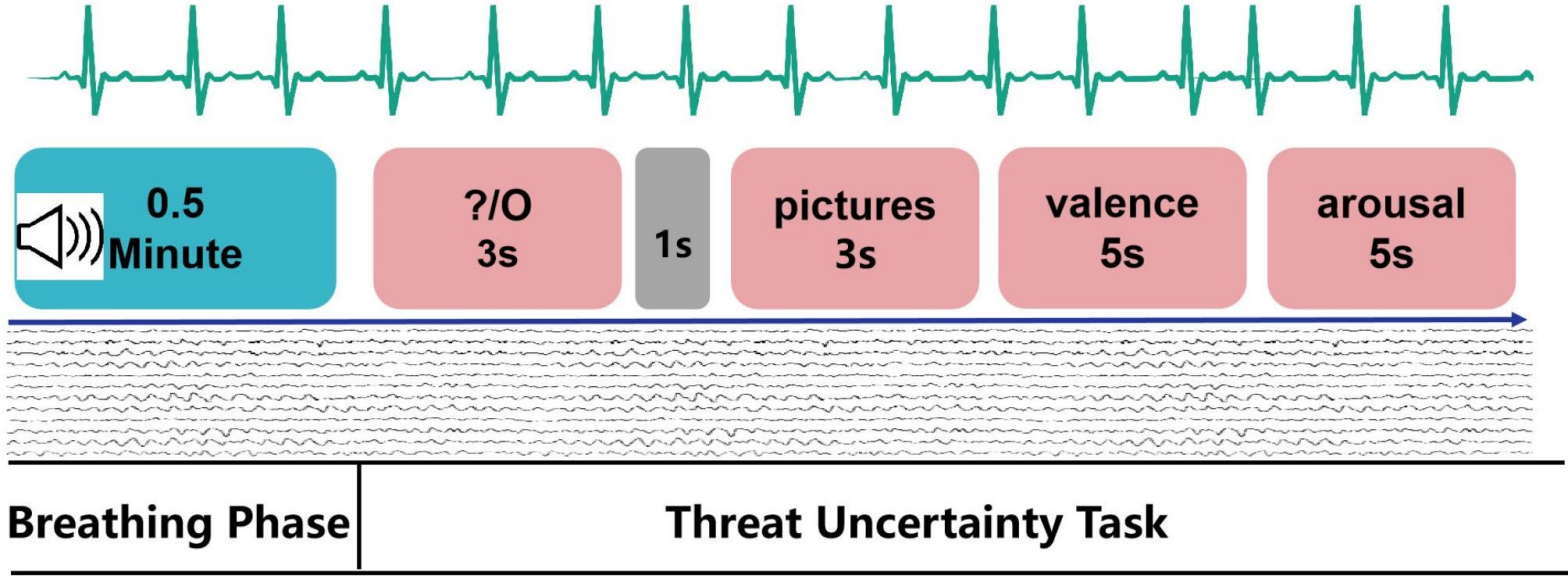
The experimental procedure of paced breathing-threat uncertainty task. After breathing exercises (the two breathing conditions were presented alternately, the fast breathing present in the first trail, the slow breathing will present in on the next trail), then, an anticipatory cue was presented, followed by the presentation of an emotionally negative stimulus, finally, the subjects rated the pictures.The gray figure indicates the blank screen.

During the threat uncertainty task, the task contained two different cue types: certain or uncertain. First, participants received a 3-second cue (“O” or “?”).To indicate the type of picture that will appear later. (certain: the “O” represented negative pictures, uncertain: “?” represented uncertainty about the type of picture to be presented, which could be neutral, positive, or negative (anticipatory anxiety: after “O” or “?”, before the picture.)) The images were shown after a 1 s blank screen, and viewing the picture for 3 s. Finally, participants evaluated its valence and arousal, with each rating allocated 5 s. participants rated the valence of the images on a scale from 0 (not negative at all) to 6 (very negative), and the arousal on a scale from 0 (not feeling at all) to 6 (very strong). In fact, in the “?”, only negative images were shown, and the subjects didn’t know it. This cycle continued until a total of 60 trials were completed. Through out the experiment, both electrocardiogram (ECG) and electroencephalography (EEG) data were collected simultaneously, and the entire procedure was managed using E-Prime 2.0 (see Fig. 1).

The 60 negative images from the International Affective Image System had a valence range of 1.26–3.07 (mean ± SD, 2.31 ± 0.41) and an arousal range of 2.50–6.89 (mean ± SD, 5.60 ± 0.63). The pictures were randomly assigned to each experimental condition. In order to ensure the equitable distribution of images, we conducted a 2 (respiratory rate: fast breathing, slow breathing) × 2 (certainty: certain, uncertain) repeated-measures ANOVA, with valence and arousal as dependent variables. The results show that whether valence or arousal is the dependent variable, the main effect of respiratory rate (*p* = 0.161) or (*p* = 0.121) was not significant, and the main effect of certainty (*p* = 0.500) or (*p* = 0.771) was not significant. The interaction between respiratory rate and determinism (*p* = 0.746) or (*p* = 0.941) was not significant. The findings show that the image distribution in each experimental condition does not influence the outcomes. Thus, the random assignment of images in this study is considered both reasonable and effective.

## Electrocardiogram and electroencephalography data acquisition, pre-processing and analysis

Acqknowledge 5.0 was used for Heart rate and heart rate variability (HRV) analysis; time and frequency domain parameters were calculated. The time domain indicator used was RMSSD (ms), namely the square root of the mean squared differences between successive RR intervals. The frequency domain index was HF (ms2), namely, the absolute power of the high-frequency band (0.15–0.4HZ). Specifically, RMSSD represents short-term variation in NN cycles and high-frequency oscillations caused by parasympathetic activity. HF is a marker of parasympathetic tone. In this study, to ensure the stability of each indicator in each experimental condition, we calculated the average value of each indicator under the same conditions for subsequent data analysis.

EEG data were collected with a 64-channel Brain Products system (Brain Products GmbH, Munich, Germany), according to the international 10–20 system. The midfrontal electrode (FCz) was used as the reference and the inion electrode (AFz) as the ground. Electrode impedance was kept below 10 kΩ for all channels. All signals were recorded with a sampling rate of 500 Hz; EEG data were pre-processed offline using EEGLAB (v2021.1;^38^) toolbox algorithms running on a MATLAB environment (R2021a, MathWorks Inc.). Two electrodes, HEO and VEO, were excluded. Then, the EEG signal was re-referenced to the average reference (i.e., calculated across all electrodes)^39^. An introduction to the event-related potential technique. MIT press.). Data were filtered by applying a high-pass filter of 0 Hz (forward-phase Butter-worth filter, 6-dB/octave roll-off) and a low-pass filter of 40 Hz (zero-phase Butterworth filter, 24-dB/octave roll-off) with an additional notch filter of 50 Hz (2 Hz width). The signal were visually inspected for the removal of artefacts and the detection of noisy channels. Bad segments were manually rejected. Noisy EEG channels were then removed and interpolated using their neighbouring channels^40,41^.

EEG data for the Spectral analysis employed the EEGLAB toolbox (^41^, http://sccn.ucsd.edu/eeglab). Spectral analysis of absolute power for 1–30 Hz was conducted using the multitaper spectral estimation with Hanning taper and 0.5 frequency resolution in EEGLAB.The spectral power was decomposed into the following spectral components: delta (0.5–3.5 Hz), theta (4.0–7.5 Hz), alpha (8.0–12.0 Hz) and beta (12.5–30.3 Hz)^42^. This spectral analysis was performed on data recorded during the breathing and emotional anticipation conditions in the whole brain.

EEG data for the Time-frequency analysis employed the EEGLAB toolbox. There are many approaches to time-frequency decomposition of EEG data, including the short-term Fourier transform (STFT), continuous or discrete wavelet transforms, Hilbert transform, and matching pursuits. In the study we used the continuous wavelet transforms (CWT) for time-frequency domain estimation. About the baseline, using a time period that is 10–20% of the overall epoch duration is recommended^43^, so the baseline time set as -500 to -200. The most common use of time-frequency analysis on EEG signals is to detect and analyze event-related synchronization / de-synchroni zation (ERS/ERD). (ERS/ERD) feature extraction uses visual detection of time-frequency regions that vary significantly from baseline. The region of interest (ROI) was selected after time-frequency analysis of EEG data. Each participant have a same ROI.The power value of the ROI is then calculated (the average of the power values at all time-frequency points within the ROI) and the calculated power value is used as the time-frequency feature of the ERS/ERD corresponding to the ROI.

## Result

### The result of self-report scores

**Fig. 2:**
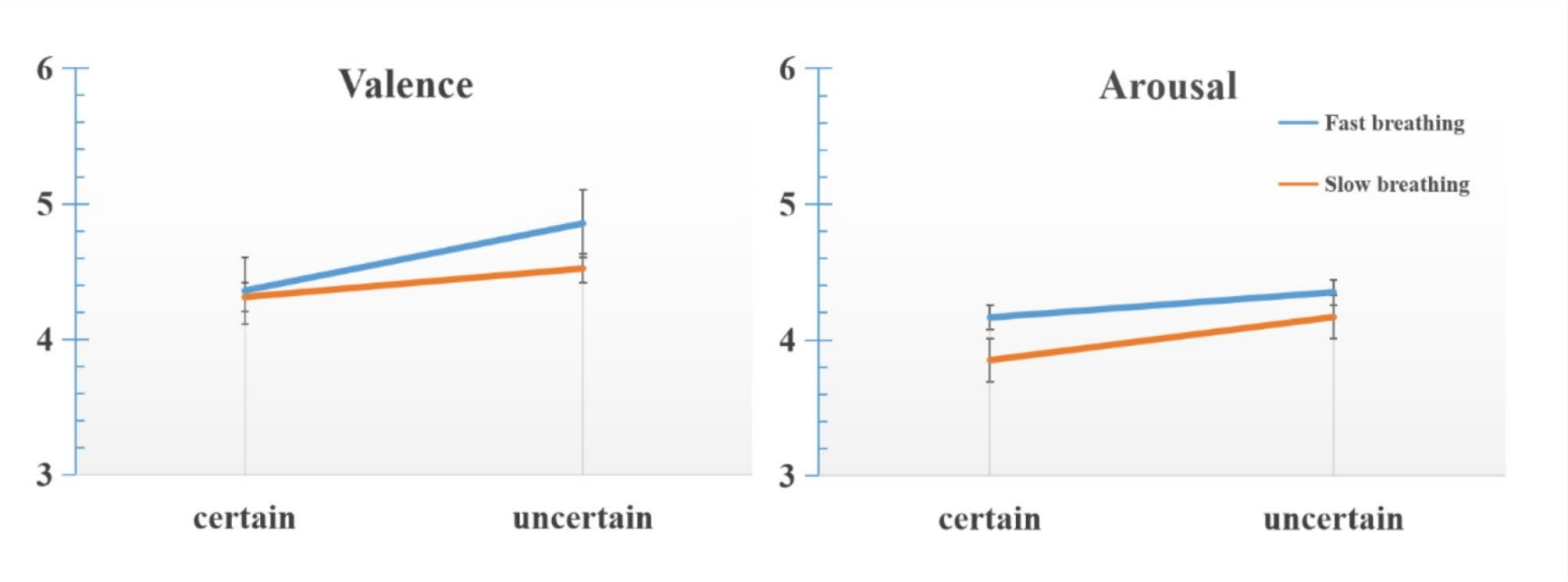
the self-report scores. The greater the value of the y-axis, the greater valence and arousal.

To investigate the impact of and certainty on anxiety, we performed an ANOVA with respiratory rate (fast breathing or slow breathing) and certainty (certain or uncertain) as the independent variables, while using valence and arousal scores as dependent variables. Tukey’s correction was applied to the analysis. All statistical analyses were performed in jamovi (v2.2.2; The jamovi project, 2021). Descriptive statistics for self-report scores are shown in Table 1; Fig. 2.

The analysis revealed a significant main effect of respiratory rate on valence (*F* = 5.46, *p* = 0.028, *η*^*2*^ = 0.023), indicating lower valence scores during slow breathing compared to fast breathing. Certainty also had a significant main effect (*F = 20.98*,*p*  = 0.001,* η*^*2*^ *= 0.073*) ,with valence scores being higher in uncertain situations than in certain ones. The interaction between respiratory rate and certainty approached significance (*F* = 4.16, *p* = 0.052, *η*^*2*^ = 0.013). Post hoc tests confirmed that the effect of respiratory rate was significant (*p* = 0.028), showing lower valence scores during slow breathing. Certainty’s effect was also significant (*p* = 0.001), with lower valence scores in certain situations.

For arousal, there was a significant main effect of respiratory rate (*F = 5.473*, *p*  = 0.028,* η*^*2*^ *= 0.018*), indicating lower scores during slow breathing. The main effect of certainty was also significant (*F = 5.940*, *p*  = 0.023,* η*^*2*^ *= 0.017*), with higher arousal scores in uncertain situations. However, The interaction between respiratory rate and certainty was not significant (*F* = 0.607, *p* = 0.443, *η*^*2*^ = 0.001). Overall, the findings suggest that certainty of success increases anxiety, while slow breathing helps to alleviate this anxiety.

**Table 1:** Descriptive statistics of self-report scores.

Respiration rate	Certainty	Valence	Arousal
		(m, σ)	(m, σ)
Fast breathing	Certain	4.36(0.68)	4.16(0.94)
	Uncertain	4.85(0.64)	4.35(0.97)
Slow breathing	Certain	4.31(0.58)	3.85(0.83)
	Uncertain	4.52(0.59)	4.16(0.78)

m = mean, σ = standard deviation.

## The result of HR and HRV

To investigate the effect of breathing on heart activity, we performed the paired sample t-tests comparisons for different cardiorespiratory features of interest. Specifically, we measured the mean HR, RMSSD and HFlog power as cardiac features during respiration.Table 2 provides a descriptive statistical analysis and the t-test comparison for two different respiration rates. All statistical analyses were performed in jamovi (v2.2.2; The jamovi project, 2021). Our findings revealed a significant variation in heart rate (HR) between fast and slow respiration, with an increase in heart rate observed during fast respiration. These findings imply a strong connection between the rate of respiration and heart rate.

**Table 2:** Descriptive statistics and test comparisons between two breathing rates.

	Fast breathing	Slow breathing	t-test fast versus slow
	(m, σ)	(m, σ)	(t, df, *p*)
HR bpm	73.6(9.66)	71.4(7.57)	(3.77, 24, < 0.001)
RMSSD (ms)	77.3(39.16)	84.9(59.44)	(-1.07, 24, 0.297)
HF power (ms^2^)	14,409(28978)	22,467(74608)	(-0.76, 24, 0.454)

### The result of spectral and Time-frequency analysis during in breathing phase

Spectral analysis was used to measure the absolute power of four frequency bands during breathing (see Table 3). We conducted a paired-sample t-test to explore the relationship between the EEG characteristics and breathing rate.All statistical analyses were performed in jamovi (v2.2.2; The jamovi project, 2021).

**Fig. 3:**
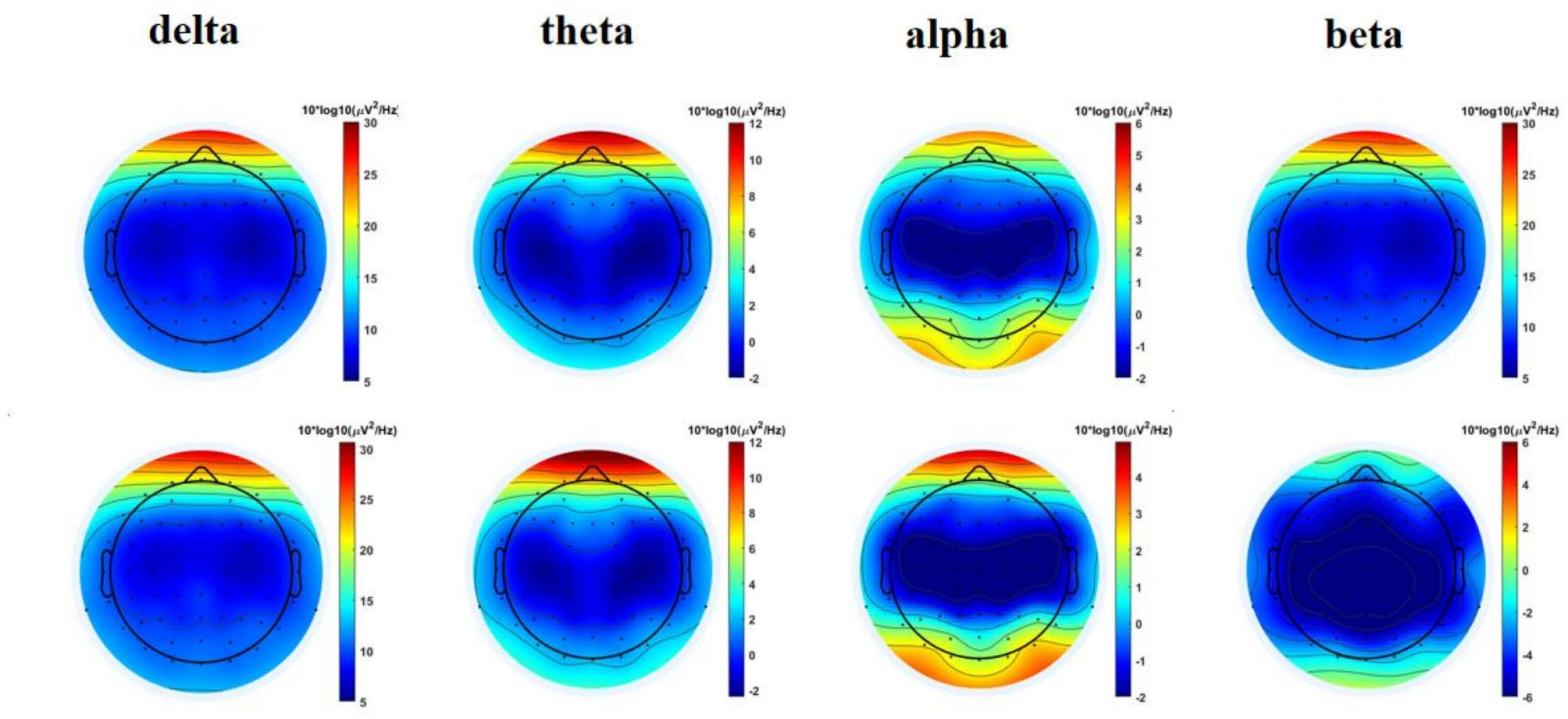
Spatial distribution map of breathing period in each frequency band; The first row shows the fast breathing, and the second row shows the slow breathing.

This test examined the total brain power during breathing, assessing the changes in the four frequency bands delta, theta, alpha, and beta during fast and slow breathing conditions (see Fig. 3). Within each frequency band, there was a significant effect by respiration rate (delta (*p <* 0.001), theta (*p <* 0.001), alpha (*p <* 0.001) and beta (*p <* 0.001)).There is an overall increase in power during slow breathing in the delta, theta, alpha and beta bands.

**Table 3:** EEG power in breathing phase.

	Delta	Theta	Alpha	Beta
	(m, σ)	(m, σ)	(m, σ)	(m, σ)
Fast breathing	10.5(1.93)	0.87(1.70)	-0.17(3.29)	-5.44(1.75)
Slow breathing	11.2(1.90)	1.38(1.68)	0.02(3.17)	-5.24(1.72)

EEG power for delta, theta, alpha and beta frequency bands (10*log10(\muV^2/HZ) (m = mean, σ = standard deviation) for breathing phase.

In order to explore the characteristics of EEG activity in the time-frequency domain at different respiratory rates, Cz was selected to analyze a respiratory cycle in the time domain (fast-breathing: 1–6 s, slow breathing: 1–13 s). Next, the region of interest (ROI) was chosen for statistical analysis (time: 0ms-500ms; frequency band: 10–15 Hz).The power value of the ROI was calculated by finding the average power value of all time-frequency points within the ROI, which was then used as the time-frequency feature of the ERS/ERD corresponding to this ROI. Each participant have a same ROI for this analysis.The average power value of the ROI under two different breathing rates was tested using a T-test and corrected by Tukey. The results indicated that there was no significant difference in time-frequency activity between the two respiratory rates. However, both respiratory rates exhibited an increase in power value in this region compared to the baseline condition, demonstrating synchronization characteristics.

### The result of spectral and Time-frequency analysis during in anticipatory anxiety phase

Using the same method mentioned above, the absolute power of the four spectral bands during the emotional anticipation phase is obtained through spectral analysis (Table 4). Next, we performed ANOVA with the absolute power of delta, theta, alpha, and beta as the dependent variables, and respiration rate (fast breathing or slow breathing) and certainty (certain or uncertain) as the independent variables. The analysis was corrected by Tukey.All statistical analyses were performed in jamovi (v2.2.2; The jamovi project, 2021).

**Fig. 4:**
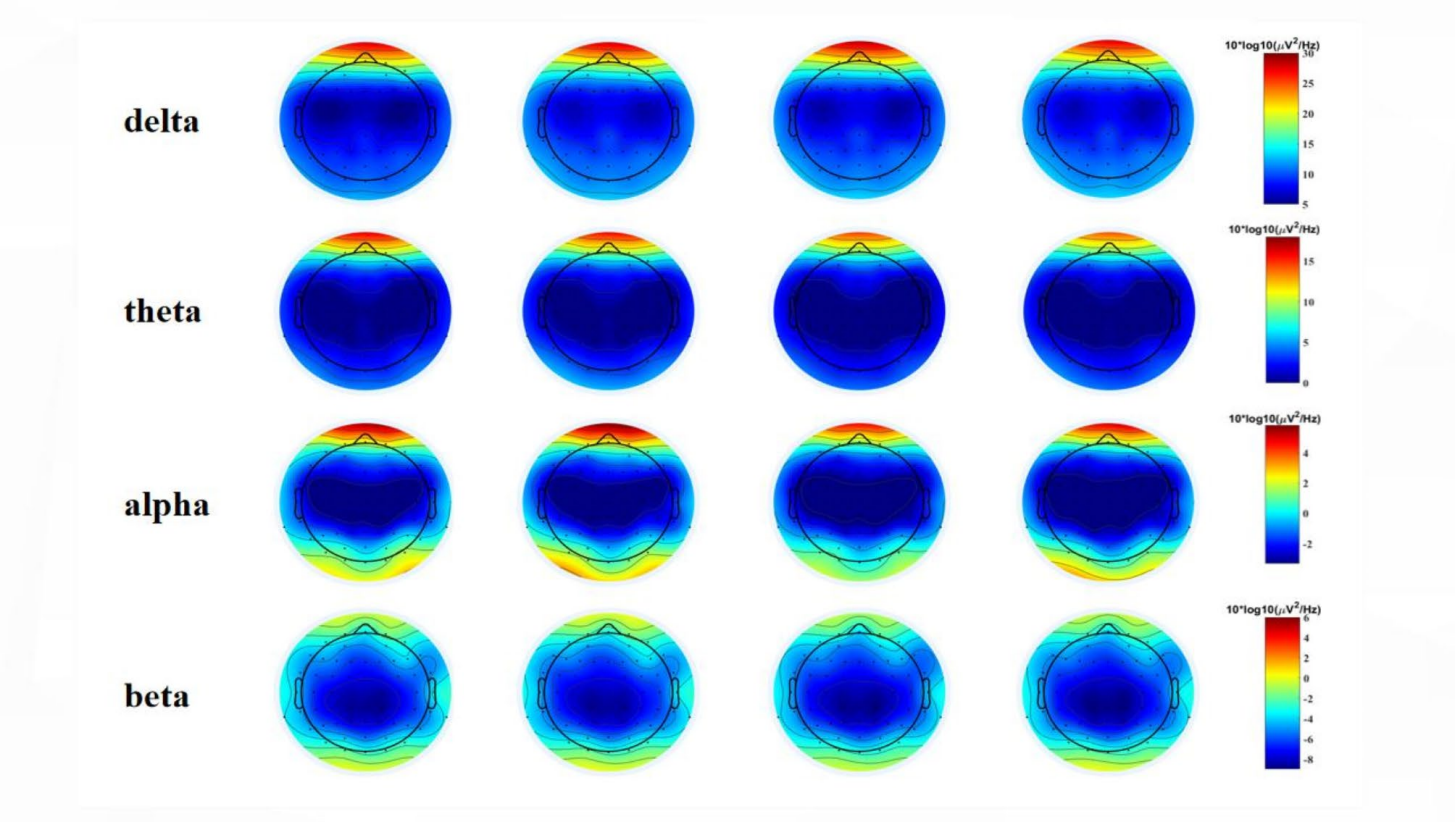
Spatial distribution map of anticipatory phase in each frequency band; The first column shows the uncertain condition of fast breathing, and the second column shows the certain condition of fast breathing. The third column is the uncertain condition of slow breathing, and the fourth column is the certain condition of slow breathing.

For the absolute power of delta (see Fig. 4), the ANOVA shows a main effect of respiratory rate (*F = 4.358*, *p*  = 0.048,* η*^*2*^ *= 0.034*), the power under fast breathing is higher than that under slow breathing. The main effect of certainty was not significant. The interaction of respiration rate and certainty was not significant .For the absolute power of theta and alpha, the ANOVA shows that the main and interaction effects of respiration rate and certainty were not significant.

For the absolute power of beta, the ANOVA shows a main effect of respiratory rate (*F = 9.95*, *p*  = 0.005,* η*^*2*^ *= 0.013*), the power under fast breathing is higher than that under slow breathing. The main effect of certainty was not significant. The interaction of respiration rate and certainty was significant (*F = 4.65*, *p*  = 0.041,* η*^*2*^ *= 0.008*). To be specific, during fast breathing, the absolute power was higher under uncertain conditions than under certain conditions. Conversely, during slow breathing, the absolute power was higher under certain conditions than under uncertain conditions. Moreover, the absolute power of theta was highest when participants were engaged in fast breathing and faced uncertainty.

In summary, only beta, The interaction of respiration rate and certainty had a significant effect on brain activity of anticipatory anxiety. When breathing slowly, the absolute power of beta decreases when facing uncertain tasks. Conversely, when breathing quickly, the absolute power of beta increases in response to uncertain tasks.

**Table 4:** EEG power in anticipatory phase.

Respiration rate	Certainty	Delta	Theta	Alpha	Beta
		(m, σ)	(m, σ)	(m, σ)	(m, σ)
Fast breathing	Certain	8.67 (8.74)	0.82 (1.37)	-0.19(2.90)	-5.45 (1.72)
	Uncertain	8.57 (1.67)	1.04 (1.34)	-0.19(2.90)	-4.99 (1.88)
Slow breathing	Certain	8.03 (1.50)	0.69 (1.49)	-0.62(2.35)	-5.54 (1.52)
	uncertain	8.09 (1.74)	0.59 (1.65)	-0.433(2.62)	-5.68 (1.73)

EEG power for delta, theta, alpha and beta frequency bands (10*log10(\muV^2/HZ) (m = mean, σ = standard deviation) for anticipatory phase.

In order to explore the influence of respiratory rate on EEG activity characteristics in the time-frequency domain of anxiety, the Cz electrode was selected to conduct time-domain analysis of EEG data in the anticipated emotional stage. The regions of interest (ROI: time: 500ms-1000ms; frequency band: 12.5 –30.3 Hz) were visually chosen for statistical analysis to observe the time-frequency characteristics. The power value of the ROI was calculated by finding the average power value of all time-frequency points within the ROI, and this calculated power value was used as the time-frequency characteristics of the ROI corresponding to ERS/ERD. Each participant have a same ROI.An ANOVA was performed with respiratory rate (fast breathing or slow breathing) and certainty (certain or uncertain) as the independent variables, and the power value of the ROI as the dependent variable. However, we didn’t find any significant results.

## Discussion

Respiratory rhythm is one of the main oscillating rhythms of the body, which is the main source of interoceptive information for the brain. Anxiety is a disease of physical and mental interaction, which is characterized by excessive worry about uncertain events in the future and disturbance of autonomic nervous system. Previous studies have explored the relationship between breathing and anxiety from low-level perceptual sensitivity and higher level metacognition^44^, but have not explored the regulating effect of interoception on anxiety at the regulatory level. This study explored the relationship between anxiety and interoception from the perspective of operable interoception-breathing, and explored the role and mechanism of slow breathing in regulating anxiety. This study combined paced breathing with the threat uncertainty task to create a new experimental paradigm for the first time. The self-reported results found that slow-paced breathing was associated with lower valence and arousal, which means that compared with fast-paced breathing rate, slow-paced breathing is a beneficial intervention response to uncertain threat information. Analysis of heart rate variability found that the heart rate was lower during slow breathing conditions. Some interesting results were found by the spectral analysis of EEG. In the breathing phase, the power of delta, theta, alpha and beta are increased during the slow-paced breathing in emotion anticipatory phase, only in beta, respiratory rate and certainty had a significant effect on the brain activity. When participants are faced with uncertain information, the beta power decreased during slow-paced breathing, however, the beta power increased during fast-paced breathing.

In the self-reported results, slow breathing was found to be more effective in reducing anxiety compared to fast breathing. We found higher levels of valence and arousal in the uncertain condition compared to the certain condition. Through slow breathing exercises, participants’ responses to the emotional stimuli of an impending uncertain threat were diminished. It may be that slow breathing prepares the individual physically and psychologically for future anxious events. Across disorders, uncertainty is thought to provoke anticipatory anxiety and to result in behaviors that are maladaptive attempts to reduce uncertainty, such as worry, reassurance seeking, checking, and hypervigilance^45^. In previous studies, uncertainty has been defined as a lack of information about an event and has been characterized as an aversive state that people are motivated to reduce. The researcher propose an uncertainty intensification hypothesis, whereby uncertainty during an emotional event makes unpleasant events more unpleasant^46^. This study supports the hypothesis. Why might online uncertainty amplify reactions to an ongoing event? One possibility is that uncertainty heightens people’s attention. That is, just as uncertainty keeps an event accessible after it occurs^47^, it might also maintain people’s attention on an ongoing event, intensifying their reactions to it. Another possibility is that uncertainty increases people’s curiosity about an emotional event, leading them to become more emotionally engaged with it. That is, people may pay equal attention to an emotional event, but those who are uncertain may be more curious about what is happening, which makes them more engaged in the event. In this study, it is confirmed that slow breathing can better regulate emotional experiences in anxious situations and expand the application of slow breathing as a convenient mode of regulation.

A higher HRV is an indicator of adequate adaptation to the new environment and effective functioning of the autonomic nervous system (ANS). The yoga practicing group showed a significant increase in HRV and a reduction in resting heart rate in a study^48^. The escalation in the heart rate is due to increased sympathetic and decreased parasympathetic activity. A significant improvement in HRV may be due to an increase in parasympathetic activity or a decrease in sympathetic activity. These factors indirectly contribute to reducing psychological parameters such as distress, anxiety, and depression in young healthy subjects^49^. In this study, the short, slow-paced breathing exercises reduced participants’ heart rate. The high spectral energy of the HRV power spectrum component reflects the activity of the parasympathetic nervous system. According to the results of Heart Rate Variability (HRV) analysis, we found that the RMSSD and HF values were higher during slow-paced breathing. This suggests that parasympathetic activity could be activated through slow breathing, effectively reducing participants’ negative emotional experiences. No significant effect of respiratory rate on RMSSD and HF was found, which may be attributed to the short duration of the breathing exercises. Previous studies have shown that the breathing exercises lasted longer than one minute. It is also worth exploring the influence of breathing on HRV from the perspective of time effect.

This study examines participants’ EEG activity during the breathing phase, revealing that slow breathing enhances neural oscillatory activity. We observed increased amplitudes in delta, theta, alpha, and beta bands during slow breathing. Other studies have also explored the effects of respiratory on EEG activity.The influence of the respiratory cycle on the EEG is also observed in the literature.During spontaneous breathing and bradypnea, there was an increase in delta power^50^. EEG studies on breathing-focused meditation show increased alpha and theta power in both the posterior and frontal regions of the brain^23,24^.The investigation of neural oscillations associated with varying respiratory frequencies during the phases of inhalation and exhalation presents a valuable area for research.Exploring the electrophysiological effects of slow breathing via EEG could shed light on the underlying neurophysiological mechanisms and aid in developing more effective strategies for managing anxiety and stress.

Over the past six years, a rapidly growing number of studies have shown that respiration exerts a significant influence on sensory, affective, and cognitive processes. At the same time, an increasing amount of experimental evidence indicates that this influence occurs via the modulation of neural oscillations and their synchronization between brain areas^51^.This study also examined the impact of slow breathing on the cognitive processing of anxiety-related cues at the neural activity level. During the anticipation anxiety phase, when participants are faced with uncertain information, the beta power increased during fast-paced breathing.High frequency oscillations of beta ranges could be considered as elementary signals of the brain, functionally related to diverse brain processes^52^ with higher power of these oscillations being a mark of higher cortical arousal. In this study, Higher breathing rates and uncertain information lead to higher levels of cortical arousal. At the same time, increased beta band activity in response to uncertain tasks may also be associated with mental effort level^53^. The elucidation of the physiological mechanisms and neural pathways regulating breathing can help to better understand how an emotional state emerges from the interaction between the body and the brain. Much work needs to be done to better delineate the direction of the relationship between breathing and anxiety, as well as to evaluate how brain systems respond to the modulation of breathing as a powerful intervention to reduce levels of anxiety.

The results of this study are encouraging. However, researchers must consider many potential shortcomings when interpreting these research results. Since it was difficult to find men to participate in this experiment, our subjects were only women, so the results cannot be generalized to men. Finally, this study only explores the regulation effect of slow breathing on anxiety, and other measures can be combined mindfulness, meditation to regulate anxiety in the future.

## Conclusion

In conclusion, the present study we combined the paced breathing task with the threat uncertainty task for the first time to investigate the role of slow breathing in regulating anxiety, and using Spectral analysis and Time-frequency domain of EEG to assess brain activity relating respiratory rate and he mechanism of respiratory rate impact on the anxious. In the study, including self- report scores, heart rate, and EEG provided evidence for the mood-regulating effects of slow breathing. These findings provide direct insight into slow breathing between anxiety, providing direct evidence that slow breathing reduces anxious.

## Data Availability

The data that support the findings of this study are available from the corresponding author upon reasonable request.
